# Maternal Characteristics Influencing the Development of Gestational Diabetes in Obese Women Receiving 17-*alpha*-Hydroxyprogesterone Caproate

**DOI:** 10.1155/2014/563243

**Published:** 2014-10-27

**Authors:** Robert Egerman, Risa Ramsey, Niki Istwan, Debbie Rhea, Gary Stanziano

**Affiliations:** ^1^Department of Obstetrics and Gynecology, University of Florida College of Medicine, 1600 SW Archer Road, P.O. Box 100294, Gainesville, FL 32610-0294, USA; ^2^University of Tennessee Health Science Center, Memphis, TN 38163, USA; ^3^Department of Clinical Research, Alere, Women's & Children's Health, Atlanta, GA 30339, USA

## Abstract

*Objective*. Gestational diabetes (GDM) and obesity portend a high risk for subsequent type 2 diabetes. We examined maternal factors influencing the development of gestational diabetes (GDM) in obese women receiving 17-*alpha*-hydroxyprogesterone caproate (17OHPC) for preterm delivery prevention. *Materials and Methods*. Retrospectively identified were 899 singleton pregnancies with maternal prepregnancy body mass indices of ≥30 kg/m^2^ enrolled for either 17OHPC weekly administration (study group) or daily uterine monitoring and nursing assessment (control group). Patients with history of diabetes type 1, 2, or GDM were excluded. Maternal characteristics were compared between groups and for women with and without development of GDM. A logistic regression model was performed on incidence of GDM, controlling for significant univariate factors. *Results*. The overall incidence of GDM in the 899 obese women studied was 11.9%. The incidence of GDM in the study group (*n* = 491) was 13.8% versus 9.6% in the control group (*n* = 408) (*P* = 0.048). Aside from earlier initiation of 17OHP and advanced maternal age, other factors including African American race, differing degrees of obesity, and use of tocolysis were not significant risks for the development of GDM. *Conclusion*. In obese women with age greater than 35 years, earlier initiation of 17OHPC may increase the risk for GDM.

## 1. Introduction

The use of progestins for the prevention of spontaneous preterm birth has regained popularity. The American Congress of Obstetricians and Gynecologists (ACOG) and the Society for Maternal Fetal Medicine Publications Committee suggest offering 17-*alpha*-hydroxyprogesterone caproate (17OHPC) administration to women with a singleton gestation having a history of a preterm birth [1]. Other studies involving shortened cervical length in an index pregnancy also give consideration for prophylactic vaginal progesterone usage [2–4]; however, current recommendations offer intramuscular 17OHP exclusively for women with a history of preterm birth [5].

While in general progesterone is known to exhibit diabetogenic properties, early reports are conflicting as to whether the use of 17OHPC increases a woman's risk for the development of gestational diabetes (GDM) [6–8]. These conflicting results may be related to differences in study design or particularly in maternal characteristics of the populations.

Rates of obesity (defined as BMI ≥ 30 kg/m^2^) continue to rise in the United States [9]. Over one-third of adult women fall within the obese category [9]. Prepregnancy obesity is a well-established risk factor for the development of GDM [10]. The purpose of this analysis was to examine maternal factors influencing the development of GDM in obese women receiving 17OHPC for the prevention of preterm delivery.

## 2. Materials and Methods

We conducted a retrospective examination of deidentified clinical information collected from high-risk pregnant women enrolled in outpatient perinatal nursing services through Alere Health between 1/2005 and 3/2009. At initiation of outpatient services, women provided written consent for the use of their deidentified protected health information for research and reporting purposes. The Women's and Children's Health Division of Alere provides comprehensive home-based services to pregnant women throughout the United States who have medical or pregnancy-related problems that could harm their pregnancies including preterm labor, gestational diabetes, hypertensive conditions, coagulation disorders, and nausea and vomiting in pregnancy.

Outpatient services were prescribed by the patient's physician as a component of the plan of care for specific conditions placing the pregnancy at risk for adverse outcomes. These nursing services were supplemental to office or clinic based care and all patient care decisions continued to be made by the primary patient care provider. Clinical data were prospectively collected from the patient and her physician throughout provision of outpatient services and at conclusion of the pregnancy and are maintained in a relational database. All outpatient data were collected using standardized definitions, forms, operating procedures, and computer systems.

The study population for this analysis consisted of obese women (prepregnancy body mass index of ≥30 kg/m^2^) with singleton gestations enrolled for outpatient services between 16 and 24 weeks of gestation and delivering at ≥29 weeks who received either 17OHPC weekly administration (study group) or daily uterine monitoring and nursing assessment for preterm labor (non-17OHPC exposed controls). Women were included if they had a complete pregnancy outcome interview and documentation of GDM diagnosis recorded as a yes or no in the database. Women with a history of type 1 or 2 diabetes or GDM in a prior pregnancy and women with a diagnosis of type 1 or 2 diabetes or GDM at enrollment for outpatient services in the current pregnancy were excluded.

Those women enrolled for 17OHPC services who received fewer than 5 weekly injections were also excluded. After inclusion and exclusion criteria, 899 women comprised the study population with 491 women in the 17OHPC exposed study group and 408 women in the non-17OHPC control group.

For the 17OHPC study group the medication was administered weekly in the patients' home by a perinatal nurse. During the weekly skilled nursing visit 250 mg of 17OHPC was given via intramuscular injection. The 17OHPC was compounded at a qualified pharmacy (PharMerica, Indianapolis, IN) using United States Pharmacopeia Convention, Inc, 〈797〉 standards in an International Organization Standardization class 5 clean room with adequate quality control procedures and documentation to assure sterility and potency of each vial. Arrangements were made for home delivery of unit dose, preservative-free vials of 17OHPC using the specifications and formulation of the 17OHPC used in the Meis et al. Network Study including the vehicle (castor oil) [11]. Similar efficacy has been demonstrated between this compounded product and the product approved by the U.S. Food and Drug Administration, Makena (Ther-Rx Corporation, St. Louis, MO) [12]. A nurse and pharmacist were available continually for patient questions and concerns.

The incidence of GDM was compared between 17OHPC exposed and nonexposed obese women. Each patient's healthcare provider made the diagnosis of GDM. The timing of GDM testing, the laboratory values used to determine the diagnosis and the GDM treatments received, were not available in the outpatient record. Similarly, the use of maintenance oral tocolytics, be they calcium channel antagonists or B agonists, was administered based on the judgment and preference of the obstetric providers and not of the authors.

Maternal characteristics were compared for those women developing GDM and those without development of GDM using Pearson's chi square, Fisher's Exact, Student's *t*-test, and Mann-Whitney *U* test statistics. A logistic regression model was performed on incidence of GDM, controlling for significant univariate factors. Two-sided *P* values were considered significant at <0.05.

## 3. Results

A total of 899 obese women met the study criteria. The overall incidence of GDM among all women studied was 11.9% (107/899). The incidence of GDM in the 17OHPC study group (*n* = 491) was 13.8% versus 9.6% in controls (*n* = 408), *P* = 0.048. Maternal characteristics of women with (*n* = 107) and without GDM (*n* = 792) are presented in Table 1. Obese women who developed GDM were more likely to be ≥35 years of age (37.4% versus 23%, *P* = 0.001) and more likely to have begun initiation of 17OHPC between 16 and 20.9 weeks (47.7% versus 36.4%, *P* = 0.024) compared to those women who did not develop GDM. No differences were noted between groups for African American race. While overall no difference was observed in the use of oral maintenance tocolytics, fewer patients in the 17OHPC group received beta agonists for tocolysis (18%) versus the control group (25.1%) (*P* < 0.002).

Logistic regression analysis was performed on the incidence of GDM controlling for advanced maternal age, length of 17OHPC exposure, and morbid obesity (Table 2). This analysis showed that longer exposure to 17OHPC and advanced maternal age influenced development of GDM in the obese women studied.

Similar rates of preterm birth at <37 weeks were identified between women with (*n* = 107) and without GDM (*n* = 792), 36.4% versus 35.4%, respectively (*P* = 0.824). Differences in rates of preterm birth at <37 weeks were observed between those in the 17OHPC group (*n* = 491) and the controls (*n* = 408), 30.5% versus 41.4%, respectively (*P* < 0.001).

## 4. Discussion

Obesity increases the risk for glucose intolerance in both nonpregnant and pregnant patients. In a retrospective analysis of obese pregnant women we compared the incidence of GDM between 17OHPC exposed and nonexposed women. Advanced maternal age as well as earlier initiation of 17OHPC in gestation led to higher frequencies of gestational diabetes.

Debate is ongoing regarding the contribution of 17OHPC to gestational glucose intolerance and gestational diabetes. Rebarber et al. retrospectively compared 557 patients receiving 17OHPC for preterm labor prophylaxis to 1524 controls who similarly had a history of preterm delivery. The frequency of gestational diabetes in the 17OHPC group was 12.9% versus 4.9% for the controls (OR 2.9 (95% CI 2.1–4.1)) [6]. Interestingly, while overall no differences were observed in the use of maintenance tocolysis, fewer patients in the 17OHPC group received beta agonists for tocolysis (18%) versus no controls (25.1%) (*P* < 0.002). As beta agonists are recognized to increase the risk for gestational diabetes and the 17OHPC group had a lower rate of exposure, we do not feel that controlling for this difference would change our results [13]. Maternal obesity was an independent risk factor for gestational diabetes as well in this study. Further, Waters et al. described the effect of 17OHPC on 110 patients and made comparisons to 330 matched unexposed controls [7]. In this retrospective analysis, the frequencies of glucose intolerance (defined as an abnormal 1 hour abnormal glucose screen) and gestational diabetes were higher in the exposed group than in the controls, 23.6 versus 11.2% (*P* < 0.001) and 10.9 versus 3.6% (*P* = 0.003), respectively. Controlling for maternal race, age, and body mass index, the odds ratio was 3.3 (95% CI 1.3–8.1) for gestational diabetes after 17OHPC prophylaxis.

Alternatively, in a secondary analysis of 2 randomized studies of 17OHPC efficacy sponsored by the Maternal Fetal Medicine Units Network, 17OHPC was not associated with higher frequencies of gestational diabetes [8]. Combining singletons and twins, 616 patients received 17OHPC and 478 were given placebo. When compared with controls, neither singleton nor twin gestations revealed greater incidences of gestational diabetes, 5.8 versus 4.7% (*P* = 0.64) and 7.4 versus 7.6% (*P* = 0.94), respectively [8]. This study found that advanced maternal age and obesity were independent risk factors for gestational diabetes; however, twin gestations, the use of 17OHPC, and African American race were not. The number of doses or time of initiation of 17OHPC was not addressed.

Progestational agents alter glucose metabolism by impairing glucose transport into the cell or by impairing insulin release [14, 15]. The degree to which administration of 17OHPC contributes to this impairment in glycemic control and detrimentally affects pregnancy remains controversial [6–8]. Modest abnormalities in maternal glucose control create increasing risk for adverse pregnancy outcomes including large birth weight, cesarean delivery, and neonatal hypoglycemia [16]. Subtleties in adverse maternal glucose control produce significant neonatal anthropomorphic changes as well as elevated cord c-peptide levels [17]. The implication for increased neonatal fat mass includes the predisposition for childhood or adult obesity.

In our study, there are several weaknesses. This was a retrospective analysis of a large data base. The diagnostic strategies for gestational diabetes including the method and timing of screening were left up to the primary obstetric provider. Since this information was obtained at the end of the pregnancy, the timing of the screening should not have influenced our results. Additionally, all patients who received progesterone received intramuscular 17OHPC in this series. Other progestational agents (progesterone gel) or mode of administration (vaginal) may have differing effects on glycemic control. Despite these limitations this study suggests that further research is needed regarding the adverse effects of prophylactic progesterone on glucose metabolism and that clinicians should remain aware that 17OHPC is a risk factor for gestational diabetes in certain high-risk groups. Progesterone has been effective at reducing preterm delivery in at-risk women who are obese. More intensive screening for glucose intolerance should be considered in these patients. Postnatally, further efforts should be made to reduce the long-term risk for the development of diabetes in these women.

## 5. Conclusion

Our observational study demonstrated that in those subjects most vulnerable to B cell dysfunction (obese women with advanced maternal age and those administered a longer course of 17OHPC) the effects of exogenous progesterone seemingly overwhelm the compensatory mechanisms of insulin production and result in a higher frequency of gestational diabetes.

## Figures and Tables

**Table 1:** Maternal characteristics of women with and without GDM (*N* = 899).

	No GDM *n* = 792	Yes GDM *n* = 107	*P* value	OR (95% CI)
AMA (≥35 years)	23.0%	37.4%	0.001	2.00 (1.31, 3.06)
African American race	37.6%	43.0%	0.284	1.25 (0.83, 1.88)
BMI 30–39.9 kg/m^2^	83.8%	76.6%	0.063	0.63 (0.39, 1.03)
BMI > 39.9 kg/m^2^	16.2%	23.4%	0.063	1.58 (0.97, 2.57)
17OHPC exposure	53.4%	63.6%	0.048	1.52 (1.00, 2.31)
17OHPC initiation				
16–20.9 weeks	36.4%	47.7%	0.024	1.59 (1.06, 2.39)
21–24.0 weeks	17.0%	15.9%	0.764	0.92 (0.53, 1.59)
Oral maintenance tocolysis	32.4%	25.2%	0.132	0.70 (0.44, 1.11)

Data presented as percentage as indicated. AMA = advanced maternal age. BMI = body mass index.

**Table 2:** Logistic regression results for adjusted risk of developing GDM.

	*P* value	OR (95% CI)
17OHPC initiation at 16–20 weeks	0.025	1.67 (1.07, 2.63)
17OHPC initiation at 21–24 weeks	0.515	1.22 (0.66, 2.26)
AMA (maternal age ≥ 35 years)	0.001	2.11 (1.37, 3.25)
Morbid obesity > 39.9 kg/m^2^	0.063	1.60 (0.97, 2.63)
